# New Enlightenment of Skin Cancer Chemoprevention through Phytochemicals: *In Vitro* and *In Vivo* Studies and the Underlying Mechanisms

**DOI:** 10.1155/2014/243452

**Published:** 2014-03-17

**Authors:** Madhulika Singh, Shankar Suman, Yogeshwer Shukla

**Affiliations:** Proteomics Laboratory, Council of Scientific & Industrial Research, Indian Institute of Toxicology Research, P.O. Box 80, M. G. Marg, Lucknow 226001, India

## Abstract

Skin cancer is still a major cause of morbidity and mortality worldwide. Skin overexposure to ultraviolet irradiations, chemicals, and several viruses has a capability to cause severe skin-related disorders including immunosuppression and skin cancer. These factors act in sequence at various steps of skin carcinogenesis via initiation, promotion, and/or progression. These days cancer chemoprevention is recognized as the most hopeful and novel approach to prevent, inhibit, or reverse the processes of carcinogenesis by intervention with natural products. Phytochemicals have antioxidant, antimutagenic, anticarcinogenic, and carcinogen detoxification capabilities thereby considered as efficient chemopreventive agents. Considerable efforts have been done to identify the phytochemicals which may possibly act on one or several molecular targets that modulate cellular processes such as inflammation, immunity, cell cycle progression, and apoptosis. Till date several phytochemicals in the light of chemoprevention have been studied by using suitable skin carcinogenic *in vitro* and *in vivo* models and proven as beneficial for prevention of skin cancer. This revision presents a comprehensive knowledge and the main molecular mechanisms of actions of various phytochemicals in the chemoprevention of skin cancer.

## 1. Introduction

Skin cancer is currently the most common type of human cancer worldwide. In the United States, the annual incidence rate of skin cancer is increasing each year, representing a growing public concern [1]. It has also been anticipated from the current records that nearly half of all Americans are susceptible to develop skin cancer at least once up to the age of 65. According to an estimate by the American Cancer Society 76,250 men and women have been diagnosed with skin cancer and 12,190 men and women could have been died (9,180 from melanoma and 3,010 from other nonepithelial skin cancers) in the United States in year 2012. Even if considerable progress has been done in developing effective skin cancer treatment modalities, skin cancer is still a global health problem of concern [2]. The main types of skin cancer are the following. (i) Basal cell carcinoma, the most common easily treated form of skin cancers and accounts for 90% of all cases. Long-term exposure to sunlight is the main cause of it. (ii) Squamous cell carcinoma is the second most common type of skin cancer. It is easily treated when found early, but in a small percentage of cases this cancer has metastasis potential. Both basal cell and squamous cell carcinoma are together known as nonmelanoma type of skin cancer. (iii) Melanoma is responsible for 75% of all skin cancer-related deaths [3] despite the fact that it accounts for less than 5% of all skin cancer cases. Approximately 65–90% of melanomas are caused by exposure to ultraviolet (UV) light [4]. (iv) Kaposi's sarcoma, a more slow-growing form of skin cancer, occurs in elderly men of Italian or Jewish ancestry and is caused by a Herpes family virus. It is an aggressive AIDS-related form affects about one third of patients with AIDS.

## 2. Cause of Skin Carcinogenesis

**Figure 1:**
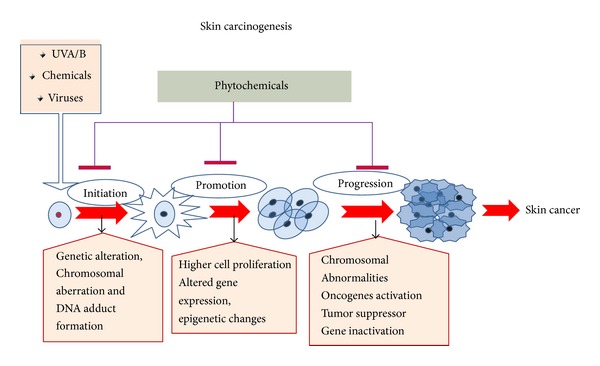
Skin cancer develops in series of events in multiple steps; however in most of studies, skin carcinogenesis is progressed in three key steps, that is, initiation, promotion, and progression, and many phytochemicals could prevent the abrupt changes in each of the steps to reverse the process of developing skin cancer.

**Figure 2:**
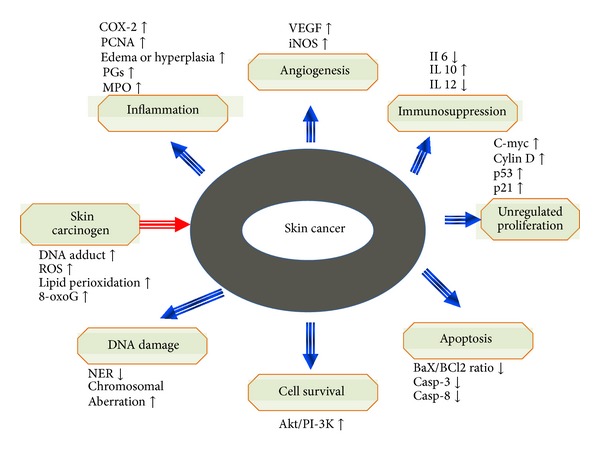
Molecular pathways are altered due to modulation of signaling pathway in skin cancer. Skin carcinogens lead to higher level of DNA adducts formation, ROS, lipid perioxidation, and 8-oxoG. Afterwards, major events like inflammation, angiogenesis, immunosuppression, higher proliferation, lesser apoptosis, enhanced DNA damage, and cell survival take place. Upregulated and downregulated molecules are depicted by arrows represented in upper and lower direction, respectively.

Skin, a major environmental interface for the body, is unintentionally or occupationally exposed to a number of harmful stimuli such as UV irradiation, viruses, and several chemical carcinogenic agents (Figures 1 and 2).

### 2.1. UV Radiation

Epidemiological data around the world have been evocative of that the exposure of skin to UV radiation is the main ecological carcinogen in the development of both melanoma and nonmelanoma types of skin cancers. UV irradiation stimulates clonal expansion of aberrant skin cells, resulting in skin carcinogenesis via involvement of multiple cellular signaling pathways [5]. The skin exposures to both ultraviolet B (UVB, wavelength 280–320 nm) and ultraviolet A (UVA, wavelength 320–400 nm) radiation have been shown to cause DNA damage and immunosuppression. In comparison to UVA radiation, UVB is more lethal and acts as complete carcinogen. It has been well recognized that UVB induced oxidative stress contributes to several adverse biological effects on the skin [6–8]. UVB is absorbed by proteins or DNA of epidermis, whereas UVA radiation penetrates deeply into the skin and reaches the lower epidermis and dermal fibroblasts. Other chromophores also absorb UV radiation to generate reactive oxygen species (ROS) and cause oxidative damage to DNA such as 8-oxo-7, 8-dihydroguanine (8-oxoG). Several tumor suppressor genes and oncogenes have been reported to be activated by UV (p53, PTCH1, BRM, and Ras) and implicated in photocarcinogenesis [9]. The alteration of the signaling molecules regulating cell proliferation, differentiation, and death is associated with UVB radiation induced skin cancer [10]. UVB radiation is known to modulate the cell cycle regulatory proteins (cyclins, cyclin-dependent kinases), antiapoptotic proteins (Akt, Bcl-2), transcription factors (AP-1, NF-*κ*B, and STAT3), proapoptotic proteins (caspases, PARP), various protein kinases (Akt, Erk, IKK, JNK, PI3K, and p38), inflammatory enzymes (COX-2), and growth factor signaling pathways (EGFR, TNF, IGF).

### 2.2. Chemicals

Over the last few years occupational skin cancer cases are reported and have mainly been owing to industrial exposure of human being to chemical carcinogens such as polycyclic aromatic hydrocarbons (PAH) and arsenic. PAH from shale oil, tar, pitch, raw paraffin, creosote, asphalt, and chimney soot have been associated with risk to skin cancers. Workers from industries in which PAH are produced (coal, coke and aluminium production plants, steel and iron foundries, and exposure to diesel engine exhaust fumes) are at higher risk of skin cancer [11]. UV radiation is reported to be a cocarcinogen to PAH induced skin cancers [12]. Arsenic in pesticides represents a potential skin cancer risk for agriculture workers. Arsenic mining and smelting industries are also place for its exposure. Being a strong mutagen, it induces large chromosomal mutations and also appears to act as a cocarcinogen with UV. Occupational coexposure to arsenic and sunlight creates an increased risk of NMSC [13].

### 2.3. Viruses

Viruses are another agent which transforms keratinocytes by activation of cancer-promoting genes. Apart from this, several viral proteins act as oncogenes which lead to the cellular proliferation. Viral skin carcinogenesis is highly concerned with immune deficient host, in such cases lower T cell reactivity and antigen presenting cells in skin assist in viral escape and tumorigenesis [14]. Among major viral agents (i) human papilloma virus (HPV) which is linked to squamous cell carcinoma (SCC) of the skin and its role in nonmelanoma skin cancer of immune competent hosts is not yet proved; (ii) Kaposi's sarcoma associated herpes virus which has wide repertoire of genes that regulate angiogenesis, inflammation, and cell cycle through which it initiates carcinogenesis; and (iii) human T-cell leukemia virus type 1 (HTLV-1) which majorly causes adult T-cell leukemia are common for skin cancer.

## 3. Animal Model for Skin Carcinogenesis Studies

In the current understanding of skin carcinogenesis, several animal models which are based on two stage carcinogenesis model framework have been developed. Among them in 7,12-dimethylbenz(a)anthracene (DMBA) or chronic UVB/A initiated and 12-O-tetradecanoylphorbol-13-acetate (TPA) promoted rodent models are widely used. The most commonly employed chemical carcinogen for inducing experimental carcinogenesis is DMBA [15]. DMBA induced experimental skin carcinogenesis is preceded by a sequence of hyperplasia, dysplasia, and carcinoma. DMBA mediates skin carcinogenesis through formation of DNA adducts, DNA damage, ROS generation, and chronic inflammation [15]. It is suggested that DMBA mediated changes are similar to those noted in human cancers [16]. DMBA initiated carcinogenesis may so be used as suitable model to study the chemopreventive potential of compounds.

## 4. Mechanistic Pathways of Skin Carcinogenesis

### 4.1. Mitogen-Activated Protein Kinases (MAPKs) Signaling Pathway

MAPKs, a family of serine/threonine-specific protein kinases belonging to the CMGC (CDK/MAPK/GSK3/CLK) kinase group [17], play a major role in translating extracellular signals into intracellular pathway through phosphorylation cascade [18]. MAPKs work mainly through the extracellular signal-regulated protein kinases (ERK), the c-Jun N-terminal kinases (JNK), and p38 kinases pathways. These are involved in regulation of cellular metabolism and gene expression and different vital actions including growth and development, disease, apoptosis, and cellular responses to external stimuli. The Raf/MEK/ERK cascade is a key signaling element in development, growth, and inflammation via the transmission of signals from cell surface receptors to the nucleus [19]. The Raf/ERK pathway is the most critical mediator of oncogene Ras-dependent carcinogenesis and UV irradiation is reported to act as inducer of it [20, 21]. Inhibition of MEK/ERK activities inhibited UV induced skin carcinogenesis [22]. In addition, Raf is one of the signaling molecules involved in the UVA induced production of matrix metalloproteinase 1 (MMP-1). MKK4, another important element of stress activated MAPK signaling pathways, phosphorylates and activates both JNK1/2 and p38 but not ERK1/2 in response to cellular stresses and inflammatory cytokines. In addition, the activation of JNK by inflammatory cytokines or by stress is frequently accompanied by the nuclear translocation of NF-*κ*B, and many genes require the concomitant activation of AP-1 and NF-*κ*B [23]. JNK1−/− mice were highly susceptible to DMBA/TPA-induced skin carcinogenesis as indicated by the increased rates of tumor growth kinetic and progression into carcinomas as compared to the wild type counterparts [24]. p38 signaling is critical for solar radiation induced skin carcinogenesis [25]. UVB irradiation results in the activation of this pathway and subsequently AP-1 activation and cyclooxygenase-2 (Cox-2) expression [26]. Hence, targeting of MAPK pathway seems as an attractive approach for the development of skin cancer chemotherapeutic agents.

### 4.2. Phosphatidylinositol-3-kinase (PI3K)/Akt Pathway

PI3K is a member of lipid kinase family and generates phosphatidylinositol-3,4,5-trisphosphate (PI(3, 4, 5)P3). PI(3, 4, 5)P3 is a second messenger essential for the translocation of Akt to the plasma membrane where it is phosphorylated and activated by phosphoinositide-dependent kinase (PDK) 1 and PDK2. Activated Akt alter the function of several substrates involved in the regulation of cell survival, cell cycle progression, and cellular growth. In recent years, it has been shown that PI3K/Akt signalling pathway components are frequently altered in cancers [27]. UV irradiation triggers the activation of EGFR and subsequently upregulates the phosphorylation of Akt, which is involved in enhanced survival of mutated cells, thereby promoting skin cancer [28]. Further, activated Akt promotes cell survival by inhibiting apoptosis by the inactivation of several proapoptotic factors Bad. The Akt level is reported to be increased during chemically induced skin carcinogenesis in mouse due to increased activity of PI3K [29, 30]. Studies have also shown that Akt regulates the activation of NF-*κ*B and AP-1 [31, 32]. PI3K has been proven to be necessary for Ras-induced malignant transformation; mice with mutations in the PI3K catalytic subunit p110*α* that block its ability to interact with Ras are highly resistant to endogenous oncogenic H-Ras-induced skin carcinogenesis [33]. Thereby, the targeting of this pathway will provide an effective means of cancer therapy.

### 4.3. The JAK-STAT Pathway

It is an important oncogenic signaling cascade that consists of the Janus kinase (JAK) family of nonreceptor tyrosine kinases and the signal transducer of activator of transcription (STAT) family of transcription factors. Under physiological conditions, the ligand-dependent activation of the JAK-STAT pathway is transient and securely regulated. The JAK family plays a critical role in cell survival, proliferation, differentiation, and apoptosis. However, in most malignancies, STAT proteins and particularly STAT3 are aberrantly activated (tyrosine phosphorylation). STAT3 regulates the expression of a variety of genes in response to cellular stimuli and thus plays a key role in cell growth and apoptosis. The activation and interaction between STAT3 and NF-*κ*B play vital roles in control of the communication between cancer cells and inflammatory cells. STAT3 and NF-*κ*B are reported as two major factors controlling the ability of preneoplastic as well as malignant cells to resist apoptosis-based tumor-surveillance and regulating tumor angiogenesis and invasiveness. Therefore, the JAK-STAT3 pathway has been suggested as playing a critical role in cell transformation and carcinogenesis. Intervention of this pathway will offer opportunities for the design of new chemopreventive and chemotherapeutic approaches.

### 4.4. Cyclooxygenase (Cox)

Derived prostaglandins (PGs) exhibit multiple functions in severe and chronic skin inflammation provoked by various physical (UV light, wounding) and chemical (TPA, arachidonic acid) injurious stimuli. COX has two main isoforms as COX-1 and COX-2. COX-1 is constitutively expressed in most tissues, whereas COX-2 is inducible by a variety of tumor promoting agents [34]. It has been observed that Cox-2 is upregulated after UV exposure in skin cells and is involved in the development of skin cancer [35]. Lack of one allele of Cox-2 mice had a 50–65% reduction of tumor multiplicity and a marked decrease in tumor size in a TPA-induced murine tumorigenesis model. Topical administration of indomethacin after each UV exposure was also effective, suggesting that a postexposure approach to skin cancer prevention may be effective [36]. Thus, the interruption of COX-2 is an effective strategy to treat and chemoprevent the skin cancer.

### 4.5. Hypoxia-Inducible Factor-1 (HIF-1)

Heterodimeric factor consisting of two subunits *α* and *β* is a master regulator of the transcriptional response of cells to oxygen deprivation. Since its discovery in the early 1990s, HIF-1 has quickly attracted interest both for its involvement in fundamental biological processes, including tumor metabolism, angiogenesis, metastasis, and inflammation, and for its potential role as therapeutic target [37]. In addition, HIF-1 can induce in an oxygen-independent manner by various cytokines through the PI3-K/Akt signal transduction pathway [38]. Deregulation of HIF-1alpha is associated with UVB-induced hyperplasia of the epidermis [39]. UV radiation induces HIF-1alpha and VEGF expression via the EGFR/PI3K/DEC1 signaling pathway [40]. Thus, developing an agent that can suppress HIF1 expression could be a critical strategy for the chemoprevention of skin cancer.

Many angiogenic factors, including vascular endothelial growth factor (VEGF) and matrixmetalloproteinases (MMPs), are also upregulated in skin cancer.

## 5. Skin Cancer Chemoprevention by Using Phytochemicals: Mechanistic Studies

**Figure 3:**
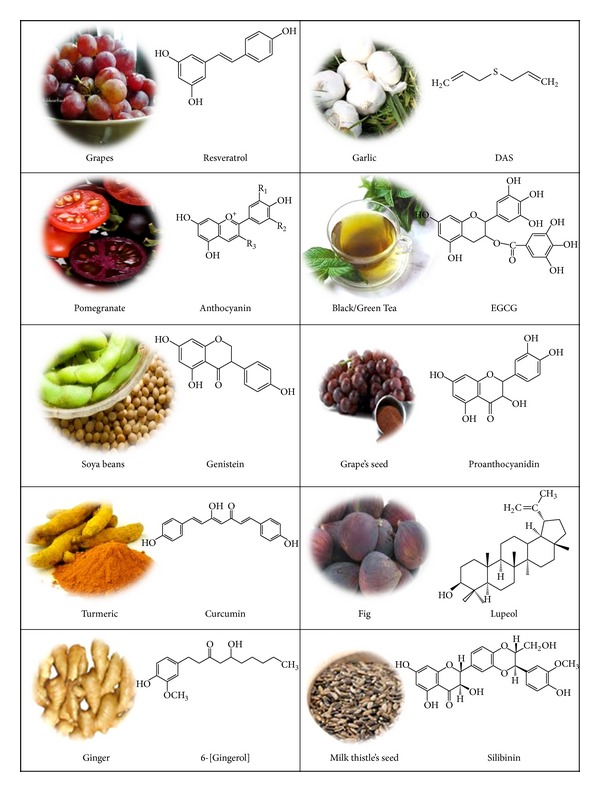
Phytochemicals and their major source associated with chemoprevention of skin cancer as discussed in text.

Cancer chemoprevention by using phytochemicals to prevent or suppress the process of carcinogenesis is still an area of active investigation. Epidemiological studies have provided persuasive evidence that phytochemicals can prevent this process. Laboratory researches have further established the effectiveness of a number of bioactive natural components that have the capacity to check cancer and other chronic diseases. Skin carcinogenesis, a multistep process, allows for the possible curative intervention; thus, cancer chemoprevention is a very promising approach to successfully achieve this goal. Lots of compounds belonging to diverse chemical classes have been identified and proved as potential skin cancer chemopreventive agents. Fruits, vegetables, seeds, flowers, leaves, and bark represent huge reservoirs of phytochemicals such as polyphenols, flavonoids, isoflavonoids, proanthocyanidins, phytoalexins, anthocyanidins, and carotenoids. Many of them have already been studied extensively for their potential anticancer or chemopreventive efficacy and some of them are at clinics now. According to estimates, on average 0.2–1 g/d of these agents are consumed as part of a regular diet [41]. Thus, the better understanding of the knowledge of underneath molecular mechanisms of cancer chemoprevention not only is important for the safe and effective use of these compounds in cancer patients but also allows for further development of novel and better therapy for cancer patients. In this review we summarized the update knowledge of photochemical reported to have skin cancer chemopreventive prospective (Figure 3 and Table 1).

### 5.1. Proanthocyanidins

Grapes (*Vitis vinifera*) are one of the most widely consumed fruits in the world and its seeds are the rich source of proanthocyanidins (oligomeric flavonoids) which are well known to exert anti-inflammatory, antioxidant, antiarthritic, and antiallergic activities, prevent skin aging, and inhibit UV radiation-induced peroxidation activity. Other rich sources of proanthocyanidins are red wine, cocoa, apples, peanuts, almonds, cranberries, blueberries, and bark of the maritime pine. Grape seed proanthocyanidins (GSPs) are promising bioactive phytomolecules that have shown antiskin carcinogenic effects and reveal no apparent toxicity* in vivo* [42–44]. The results of acute and subchronic toxicity testing in rats,* in vitro* genotoxicity testing in mammalian cell, and* in vivo* mouse micronucleus test reveal a low toxicity and no genotoxic potential of proanthocyanidins-rich extract from grape seeds [45]. The bioavailability of proanthocyanidins is largely influenced by their degree of polymerization. The absorption rate of proanthocyanidin dimers is 5–10% of that of (−)-epicatechin [46]. Trimers and tetramers had lower absorption rates than dimers. Absorbed intact dimers, trimers, and tetramers undergo limited phase II metabolism in the intestine and liver in rats [46]. Employing HPLC nine individual polyphenols is identified in GSPs as catechin, epicatechin, procyanidins B1–B5 and C1, and procyanidin B5-3′-gallate which are potent antioxidant in terms of inhibition of epidermal lipid peroxidation [47]. Intake of GSPs prevents skin cancer through their anti-inflammatory, antioxidative, and DNA repair mechanisms in both UV radiation-induced and DMBA-initiated/TPA-promoted mouse skin tumors. Mittal et al. [42] reported that dietary intake of GSPs (0.2 and 0.5%, w/w) resulted in prevention against UVB-induced complete initiation and promotion stages of photocarcinogenesis in terms of tumor incidence (20–95%), multiplicity (46–95%), and size (29–94%). Treatment of GSP significantly inhibited UVB or Fe3+ induced lipid peroxidation by 57–66% (*P* < 0.01) and 41–77% (*P* < 0.05–0.001), respectively, thus suggesting the strong antioxidant mechanism of photoprotection offered by GSPs [42]. According to Sharma et al. [48, 49] same doses of GSPs have the ability to protect the skin from the adverse effects of UVB radiation via modulation of the MAPK and NF-*κ*B pathways. GSPs inhibited UVB-induced infiltration of proinflammatory leukocytes and the levels of myeloperoxidase (MPO), Cox-2, PGE2, Cyclin D1, and PCNA in the skin and skin tumors compared to non-GSPs-treated UVB irradiated counterpart [50]. GSPs reduced the UVB-induced increase in immunosuppressive cytokine interleukin (IL-) 10 in skin and draining lymph nodes compared with mice that did not receive GSPs. In contrast, GSPs enhanced the production of immune stimulatory cytokine IL-12 in the draining lymph nodes. GSPs fed mice exhibit a reduction in UV-induced suppression of allergic contact hypersensitivity, a prototypic T-cell-mediated response [51]. Results suggest that GSPs prevent UVB-induced immunosuppression through DNA repair dependent functional activation of dendritic cells and reexpression of tumor suppressor genes* RASSF1A*,* p16INK4a*, and* Cip1/p21*. Skin painted with GSPs before UV radiation showed fewer sunburn cells and mutant p53-positive epidermal cells and more Langerhans cells compared with skin treated with UV radiation only [52]. Dose dependent antitumor-promoting effects of GSPs were evident in terms of a reduction in tumor incidence (35 and 60% inhibition), multiplicity (61 and 83% inhibition), and volume (67 and 87% inhibition) at both 0.5 and 1.5 mg GSP, respectively, in DMBA/TPA treated SENCAR mouse skin model [48]. Pretreatment of CD-1 mouse skin with GSP (5–30 mg doses) resulted in a dose-dependent reduction in TPA-induced ODC and MPO activity [53, 54].

Mantena and Katiyar [54] attempted to define the photoprotective mechanism of action of GSPs. Researchers observed that* in vitro* treatment of NHEK with GSPs resulted in the prevention of UVB-induced depletion of antioxidant defense enzymes (GPx, catalase, SOD, and GSH) and H_2_O_2_ production. Further, GSPs inhibit H_2_O_2_-induced phosphorylation of ERK1/2, JNK, and p38 proteins. Meeran and Katiyar [55] reported that GSPs inhibited skin cancer cell (A431 cells) proliferation which was mediated through the inhibition of cyclin-dependent kinases (Cdk) Cdk2, Cdk4, and Cdk6 and cyclin D1, D2, and E, increase in cyclin-dependent kinase inhibitors (Cdki), Cip1/p21 and Kip1/p27, and enhance binding of Cdki-Cdk. GSPs have the ability to inhibit highly metastasis-specific human melanoma cells invasion/migration by targeting the endogenous Cox-2 expression and PGE_2_ production and reversing the process of epithelial-to-mesenchymal transition [56]. The precise epigenetic molecular mechanisms of anticancer potential of GSPs have been explored by Vaid et al. [57]. Treatment of skin cancer cells with GSPs decreased the levels of global DNA methylation, 5-methylcytosine, DNA methyltransferase (DNMT) activity, and mRNA and protein levels of DNMT1, DNMT3a, and DNMT3b in treated cells.

### 5.2. Tea Polyphenols

Tea (*Camellia sinensis*; Theaceae) has been consumed as a popular beverage worldwide and skin photoprotection by green tea polyphenols (GTPs) has been widely investigated. Besides health beneficial effects toxicity of tea polyphenols is attributed to their role as prooxidants in the presence of redox-active chemicals and lead to the formation of reactive oxygen species which can cause damage to DNA, lipids and other biological molecules [77, 78]. Oral feeding of a GTP or water extract of green tea affords protection against UVB radiation induced inflammatory responses and carcinogenesis in animals [79]. GTP (0.2% w/v) as the sole source of drinking water for 30 days to SKH-1 hairless mice followed by irradiation with UVB (900 mJ/cm^2^) offered a significant protection against UVB radiation induced cutaneous edema, epidermal ODC and Cox-2 activities, and depletion of the antioxidant-defense system in epidermis [80]. Further elevated levels of nucleotide excision repair (NER) genes show a novel mechanism by which drinking GTPs prevents UV-induced immunosuppression [81]. Prevention of photocarcinogenesis by GTPs is mediated through IL-12-dependent DNA repair and a subsequent reduction in skin inflammation [82]. GTPs (0.2%, w/v) in drinking water significantly reduced UVB-induced tumor development, markers of inflammation (Cox-2, PGE2, PCNA, and cyclin D1) and proinflammatory cytokines (TNF-*α*, IL-6, and IL-1*β*) in wild-type mice, when compared with IL-12-knockout mice. Topically repeated doses of GTP (5 mg/mouse) resulted in significant decrease in UVB (180 mJ/cm^2^) induced bi-fold-skin thickness, skin edema and infiltration of leukocytes, and modulations in MAPK and NF-*κ*B signaling pathways [83]. Application of epigallocatechin gallate (EGCG) protects against radiation induced immunosuppression by tolerance induction, blocking the migration of antigen-presenting cells from the skin to draining lymph nodes (DLN) and markedly inducing IL-12 dependent DNA repair mechanism [81, 84–86]. In addition EGCG decreased the number of UVB-induced increases in H_2_O_2_-producing cells and inducible nitric oxide synthase (iNOS-) expressing cells and the production of H_2_O_2_ and NO in both exposed part of epidermis and dermis [87]. Findings of a study done by Vayalil et al. [88] suggest the long-term photopreventive effects of EGCG as a cream based formulation of GTP which was for the first time tested in this study to explore the possibility of its use for the humans. Topical application of EGCG (1 mg/cm^2^) in hydrophilic ointment before single (180 mJ/cm^2^) or multiple (180 mJ/cm^2^, daily for 10 days) doses of UVB exposure resulted in prevention of UVB-induced depletion of antioxidant enzymes levels. Further, treatment of EGCG to mouse skin resulted in marked inhibition of a single UVB induced phosphorylation of ERK1/2 (16–95%), JNK (46–100%), and p38 (100%) proteins in a time-dependent manner. Photoprotective efficacy of GTP given in drinking water (0.2%, w/v) was also compared with that of topical treatment of EGCG and GTP by Vayalil et al. [88]. It was noted that lesser photoprotective efficacy of GTP in drinking water in comparison with topical application may be due to its less bioavailability in skin target cells. Pretreatment with EGCG was also found to restore the UV-induced decrease in GSH level and afforded protection to the antioxidant enzyme GPx in human skin [87].

Topical application of black tea polyphenols (BTP) (6 mg/0.2 mL acetone/animal) 30 min prior to TPA application on to the mouse skin resulted in inhibition against TPA-induced epidermal edema, hyperplasia, leukocytes infiltration, and induction of epidermal ODC and proinflammatory cytokine IL-1alpha mRNA expression and Cox2 activities [89]. Thearubigins or polymeric black tea polyphenols (PBPs) have been shown to possess antitumor-promoting effects in two-stage skin carcinogenesis. Pretreatment with PBP fractions exhibits antitumor promoting effects as evidenced by differentially altered latency, multiplicity, and incidence of skin papillomas [90]. PBPs pretreatment decreased TPA-induced translocation of PKC isozymes (*α*, *β*, *η*, *γ*, and *ε*) from cytosol to membrane and PKC phosphorylation [88]. These antipromoting effects of PBPs are due to modulation of TPA-induced PI3K-mediated signal transduction [91]. PBPs decreased TPA-induced cell proliferation by decreasing the activation of signaling kinases (c-Jun, ERK, p38, and Akt), transcription factors (AP-1 and NF-*κ*B), and inflammatory protein (Cox2) [90].

*In vitro* doses of EGCG to NHEK cells before UVB exposure inhibits UVB-induced H_2_O_2_ production and activation of MAPK [92] and NF-*κ*B pathways [90]. EGCG treatment resulted in a dose-dependent inhibition of cell growth and induction of apoptosis mediated through NF-*κ*B inhibition in A431 cells but not in normal cells [93, 94]. Recent findings suggest that GTP reduce skin cancer cells survival by influencing polycomb group proteins (PcG-) mediated epigenetic regulatory mechanisms [95, 96]. EGCG and DZNep, independently and in combination, reduce the level of PcG proteins, namely, Ezh2, eed, Suz12, Mel18, and Bmi-1 [95]. This change in PcG protein expression was associated with reduced expression of cyclin-dependent kinases (cdk) (1, 2, and 4), cyclin (D1, E, A, and B1), and increased expression of cell-cycle inhibitory proteins (p21 and p27) [93]. Apoptosis was evidenced by increased cleavage of caspases (9, 8, and 3) and increased cleavage of PARP [95].

A population-based, case-control study conducted on 450 individuals showed that tea concentration specifically black tea, brewing time, and beverage temperature has major influences on the potential protective effects of tea in relation to skin squamous cell carcinoma [97]. According to Bickers and Athar, topical application of tea extracts to human volunteers protects against UVB-induced erythema [97]. These studies indicate that tea extracts are effective in reducing UVB- and PUVA-mediated DNA damage and expression of early response genes and early inflammatory changes in human skin [98]. Katiyar et al. in year 2000 conducted a human study and reported that topical treatment with GTP (~1 mg/cm^2^ of skin area) 20 min before human buttock skin (sun-protected site) exposure to UVB inhibited CPD formation in epidermis by 81, 70, 60, and 60% at 0.5, 1.0, 2.0, and 4.0 minimal erythema dose of UV exposure, respectively [99]. Further, treatment of human skin with varying doses of GTP (1–4 mg/2.5 cm^2^ of skin area) before a single dose of UVB exposure (4.0 minimal erythema dose) decreased dose dependently the formation of UVB-induced CPDs in both epidermis and dermis.

### 5.3. Pomegranate Fruit Extract

Pomegranate fruit (*Punica granatum *L.) possesses extraordinary medicinal properties and has been named as the “nature's power fruit” since ancient time. Traditionally pomegranate including its roots, tree bark, fruit juice, leaves, and flowers has been used to treat several ailments in human.* P. granatum* contains anthocyanins (delphinidin, cyaniding, and pelargonidin) and hydrolyzable tannins (punicalin, pedunculagin, punicalagin, and gallagic and ellagic acid esters of glucose), reported to have strong antioxidant and anti-inflammatory properties [100]. Pomegranate fruit extract (PFE) possesses antitumor promoting effects in a mouse model of skin carcinogenesis induced by chemicals as well as UV radiations [101, 102]. Application of PFE (2 mg/mouse) prior to TPA on CD-1 mouse skin afforded significant inhibition in TPA-induced increase skin edema and hyperplasia, epidermal ODC activity, and protein expression of ODC and Cox-2 [103]. Further, PFE resulted in inhibition of TPA-induced activation of ERK1/2, p38, JNK1/2, NF-*κ*B, and IKK*α* proteins. The effects of skin application of pomegranate seed oil and PFE on two stage skin carcinogenesis model (DMBA-initiated and TPA-promoted) were also evaluated by the same researchers [103, 104]. Pomegranate seed oil (5%) and PFE application prior to TPA on mouse skin resulted in delay in latency period from 9 to 14 weeks and afforded considerable protection in terms of tumor incidence and tumor multiplicity [103, 104]. Feeding of PFE (0.2% w/v) for 14 days inhibited UVB-induced skin edema, hyperplasia, infiltration of leukocytes, lipid peroxidation, H_2_O_2_, ODC activity, and expression of ODC, Cox-2, PCNA iNOS, cyclin D1 and MMP-2, MMP-3 and MMP-9 proteins in SKH-1 mice [105, 106]. PFE supplemented diet also enhanced the repair of UVB-mediated formation of both CPDs and 8-oxodG [105] and these events were associated with PFE induced inhibition of MAPK and NF-*κ*B pathways [105, 106].

*In vitro* treatment of PFE (10–40 *μ*g/mL) also protects against the adverse effects of UVB (40 mJ/cm^2^) radiation by inhibiting UVB-induced modulations of NF-*κ*B and MAPK pathways and provides a molecular basis for the photochemopreventive effects of PFE [107]. Treatment of NHEK cells with PFE (60–100 *μ*g/mL for 24 h) prior to UVA exposure resulted in a dose-dependent inhibition of UVA-mediated phosphorylation of STAT3 at Tyr705, AKT at Ser473, ERK1/2 and mTOR at Thr2448, and p70S6K at Thr421/Ser424 [108]. Further, PFE pretreatment of NHEK was found to increase the G1 phase cell-cycle arrest and the expression of Bax and Bad with downregulation of Bcl-xL [108]. Pretreatment of PFA (10–40 *μ*g/mL) protects HaCaT cells against UVB (15–30 mJ/cm^2^-) induced oxidative stress and markers of photoaging and could be a useful supplement in skin care products [109]. In a study, Pacheco-Palencia et al. [110] investigated potential protective effects of a PFE against both UVA and UVB-induced damage in human skin fibroblast (SKU-1064) cells. PFE (5 to 60 mg/L) was effective in protecting UV induced cell death and likely related to a reduced activation of NF-*κ*B, a downregulation of caspase-3, and an increased G0/G1 phase. Authors further suggested that higher polyphenolic concentration (500–10000 mg/L) is needed to achieve a noteworthy reduction in UV-induced ROS levels.

Afaq and colleagues [111] determined the effects of pomegranate-derived products, POMx juice, POMx extract, and pomegranate oil (POMo), against UVB-mediated damage using reconstituted human skin (EpiDerm(TM) FT-200). Pretreatment of Epiderm resulted in inhibition of UVB-induced CPD, 8-OHdG, protein oxidation, and expression of collagenase (MMP-1), gelatinase (MMP-2, MMP-9), stromelysin (MMP-3), marilysin (MMP-7), elastase (MMP-12), tropoelastin, c-Fos, c-Jun, and PCNA. Collectively, these results suggest that all three pomegranate-derived products may be useful against UVB-induced damage to human skin.

A double-blind, placebocontrolled trial to clinically evaluate the protective and ameliorative effects of ellagic acid-rich pomegranate extract on pigmentation in the skin after UV irradiation, using female subjects of age 20–40 s, was performed by Kasai et al. [112]. Ellagic acid-rich pomegranate extract ingested orally by the subjects has an inhibitory effect on a slight pigmentation in the human skin caused by UV irradiation, suggesting the relevance of laboratory data in human too.

### 5.4. Resveratrol

Resveratrol, a trans-3,5,4′-trihydroxy-trans-stilbene, is a phytoalexin, produced by many plants and the skin of red grapes exhibits versatile biological effects. Besides its protection of the cardiovascular system, it can affect the essential stages of carcinogenesis, involving tumor initiation, promotion, and progression. Topical doses of resveratrol either pre- or post- UVB irradiation has been tested for its efficacy against the development of skin cancer. Topical doses of resveratrol to SKH-1 hairless mice significantly inhibited single or multiple doses of UVB (180 mJ/cm^2^-) mediated phototoxicity as evidenced by reversal of bi-fold skin thickness, skin edema, and hyperplasia [113]. Similarly, in mouse skin DMBA-TPA model, treatment with resveratrol doses showed up to a 98% reduction in skin tumors [114] and 60% reduction in papillomas [115]. These effects were related to its cytotoxic and free radical scavenging activities [116]. According to Fu et al, [117] diet containing resveratrol shown to inhibit DMBA/croton oil-induced skin papillomas correlated with prolonging tumor latency period and inhibiting croton oil-induced epidermal ODC activities. Resveratrol doses in SKH-1 mice skin decreased the expression of cyclins D1 and D2, Cdk 2, 4, and 6, and PCNA and increased expression of p21WAF1/CIP [118]. Further, activation of p53 and Bax along with reduced expression of antiapoptotic proteins (survivin and Bcl-2) and markers of tumor promotion (Cox-2 and ODC) was observed [119, 120]. Kundu et al. [121] findings suggest that resveratrol targets IKK in blocking TPA-induced NF-*κ*B activation and Cox-2 expression in mouse skin. In a study by Roy et al. [122] chemopreventive properties of resveratrol in DMBA induced mouse skin tumors were also reflected by delay in onset of tumorigenesis, reduced cumulative number of tumors, and reduction in tumor volume. DMBA suppressed (p53, Bax, and Apaf-1) and increased (Bcl-2 and survivin) expression of proteins were modulated by resveratrol treatment. Furthermore, resveratrol supplementation resulted in regulation of PI3K/AKT pathway [122]. Results of a study by Yusuf et al. [123] indicate that toll-like receptors (TLR4) are an important mediator of resveratrol induced chemoprevention in DMBA skin tumorigenesis. Resveratrol combinations with ellagic acid, grape seed extract, and other phytochemicals are very potent inhibitors of DMBA induced skin tumorgenesis [124].

In the normal skin keratinocytes, resveratrol doses (5, 10, and 25 *μ*M) blocked UVB-mediated (40 mJ/cm^2^) activation of NF-*κ*B, phosphorylation and degradation of I*κ*B*α*, and activation of IKK*α* [125].* In vitro* data demonstrate that resveratrol induces G1-phase cell-cycle arrest accompanied by p21WAF1/CIP1 induction and inhibition of MEK1, ERK1/and AP-1 signalling in A431 cells [126–128]. Resveratrol doses arbitrated TGF-*β*2 downregulation; this event appears to occur through the inhibition of both TGF-*β*2/Smad dependent and independent pathways and thus suppressed the invasiveness of A431 cells [129]. Through the establishment of A431 cells xenografts in nude mice, Hao et al. [130] noted that the anticancer mechanism of resveratrol was through inducing apoptosis as it altered p53 and survivin expression. In a study, Bastianetto et al. [131] aimed to investigate the possible existence of specific binding sites in skin that mediate the protective action of resveratrol. Using human skin tissue, researcher reported here the presence of specific [(3)H]-resveratrol binding sites (K(D) * *=* * 180 nM) that are mainly located in the epidermis. Cottart et al. reviewed the bioavailability and toxicity of resveratrol; it seem to be well tolerated and without any marked toxicity [132]. These data are important in the context of human effectiveness studies, and authors provide further support for the use of resveratrol as a pharmacological drug in human medicine.

*Pterostilbene (trans-3,5-dimethoxy-4-hydroxystilbene)*, a natural analogue of resveratrol, found in blueberries and grapes is a powerful natural antioxidant. Tsai et al. [133] for the first time suggested pterostilbene as a novel functional agent capable of preventing inflammation-associated skin tumorigenesis which functions by downregulating inflammatory iNOS and Cox-2 gene. Pterostilbene significantly inhibited DMBA/TPA-induced skin tumor formation via suppressing activation of NF-*κ*B, AP-1, ERK1/2, JNK1/2, and PI3K/Akt.

### 5.5. Silymarin

Since long, silymarin and its major constituent silibinin, isolated from the medicinal plant Silybum marianum (commonly called as milk thistle), have been used for the treatment of hepatic ailments. Recently, these orally active flavonoids have also been interrogated for their significant anticancer effects in a variety of* in vitro* and* in vivo* systems of carcinomas including skin. Notably, it is well endured at doses up to 1% w/w or 750 mg/kg body wt in diet and showed no adverse effects to mice [134]. Topical treatment of silymarin inhibited DMBA-initiated and several tumor promoters like TPA, mezerein, benzoyal peroxide, and okadaic acid induced mouse skin carcinogenesis [135]. Mechanism of such effects involves inhibition of promoter-induced edema, hyperplasia, cell proliferation, and oxidative stress [135]. Application of silymarin (3, 6, and 12 mg/animal) prior to TPA resulted in a great protection against tumor promotion in DMBA initiated mouse skin and was apparent in terms of reduction in tumor incidence, multiplicity, and volume [135]. Singh et al. [136] demonstrated the mechanistic rationale of silymarin against skin tumors in* in vivo* study. In this study DMBA-TPA established that skin papilloma/tumor-bearing SENCAR mice were fed with 0.5% silymarin and both tumor growth and regression were monitored during entire feeding schedule. Silymarin feeding significantly inhibited tumor growth, decreased proliferation index, increased apoptotic index, and decreased phospho-ERK1/2. In UVB-induced carcinogenesis the effect of silymarin was also profound in terms of tumor incidence, multiplicity, and tumor volume [137]. Results from another short term experiments suggested that silymarin application resulted in inhibition of UVB-caused sunburn and apoptotic cell formation, skin edema, depletion of catalase activity, and induction of Cox and ODC activities and ODC mRNA expression [137]. Preventive efficacy of silibinin against UVB induced photocarcinogenesis involves the inhibition of DNA synthesis, cell proliferation, and cell cycle progression and an induction of apoptosis [138, 139]. Dietary feeding of silibinin to mice before UVB irradiation affords strong protection against UV-induced damage in epidermis via decrease in thymine dimer positive cells and increase in p53-p21/Cip1 proteins expression [139–141]. Silibinin also showed a strong phosphorylation of ERK1/2, JNK1/2, and p38 MAPK but inhibited Akt [140]. Topical treatment of silymarin (1 mg/cm^2^) inhibited UV-induced (90 mJ/cm^2^) oxidative stress through inhibition of infiltrating CD11b+ cells (showing higher production of ROS in both epidermis and dermis than CD11b-cells) in C3H/HeN mice [142]. The inhibitory effect of silymarin, in spite of whether it is given before or after UV irradiation, was of similar degree [141]. Silibinin treatment (50 mg/kg/day for 4 days) offered UVB induced improper autophagy intervention as evidenced by reversed dermal and epidermal autophagy levels [142, 143].

Anticancer and cancer preventive efficacy of these compounds are also apparent by* in vitro* studies. Treatment of skin cancer A431 cells by silibinin resulted in cell growth inhibition and death, which was found to be coupled with a decrease in MAPK/ERK1/2 levels and an upregulation of SAPK/JNK1/2 and p38 MAPK activation [136]. Studies suggest that both silymarin and silibinin are equally beneficial in the removal of UVB and UVA damaged skin cells. Pretreatment inhibited UV-induced apoptosis in HaCaT cells [144]. Following the doses of silibinin (500 *μ*M) the expression of Fas-associating protein with death domain was totally abolished and was associated with inhibition of cleavage of procaspase-8, decreased release of cytochrome c, and reduced expression of inhibitor of caspase-activated DNase and PARP. In a study by Roy et al. [145] silibinin pretreatment accelerated the repair of CPD induced by moderate dose of UVB (50 mJ/cm^2^) and p53-mediated upregulation of GADD45*α* is suggested as key mechanism. Treatment of UVA irradiated HaCaT (20 J/cm^2^) with silymarin (0.7–34 mg/L, 4 h) resulted in concentration-dependent diminution of UVA-caused oxidative stress [146]. Silymarin application extensively reduced formation of DNA single strand breaks, ROS production, lipid peroxidation, GSH depletion, and caspase-3 activity in irradiated cells. Silibinin (75 *μ*m 2 h before UVA exposure) enhances ER stress-mediated apoptosis in HaCaT cells by increasing the expression of CHOP protein [147].

### 5.6. Lupeol

Lupeol, a phytosterol and triterpene, is widely found in edible fruits and vegetables. Extensive research over the last few years has revealed several noticeable pharmacological activities of lupeol. Studies suggest that lupeol has a potential to act as an anti-inflammatory, antimicrobial, antiprotozoal, antiproliferative, anti-invasive, antiangiogenic, and cholesterol lowering agent. Effects of lupeol on TPA-induced markers of skin tumor promotion were evaluated [148]. Topical application of lupeol (1-2 mg/mouse) prior to TPA onto the skin of CD-1 mice afforded significant inhibition against TPA-mediated increase in skin edema and hyperplasia, epidermal ODC activity, and protein expression of ODC, Cox-2, and nitric oxide synthase (NOSs). The animals pretreated with lupeol showed significantly reduced tumor incidence, tumor burden, and delay in the latency period for tumor appearance. Further, lupeol doses resulted in the inhibition of TPA-induced activation of PI3K, phosphorylation of Akt, activation of NF-*κ*B and IKK*α*, and degradation and phosphorylation of I*κ*B*α* [149]. Both pre- and posttreatment of lupeol (200 *μ*g/mouse) showed significant (*P* < 0.001) preventive effects in DMBA induced DNA strand breaks in dose and time dependent manner [148]. Nigam et al. [150] demonstrated the effects of lupeol on DMBA-induced alterations in mouse skin. Cell-cycle analysis showed that lupeol-induced G2/M-phase arrest mediated through inhibition of the cyclin-B-regulated signaling pathway involving p53, p21/WAF1, cdc25C, cdc2, and cyclin-B gene. Lupeol induced apoptosis was associated with upregulation of bax and caspase-3 genes and downregulation of bcl-2 and survivin genes.

*In vitro* apoptosis inducing effects of lupeol were studied in A431 skin carcinoma cells by Prasad et al. [151]. Lupeol-induced apoptosis was associated with caspase dependent mitochondrial cell death pathway through activation of Bax, caspases, and Apaf1, decrease in Bcl-2, and subsequent cleavage of PARP.

### 5.7. Curcumin

Curcurmin (diferuloylmethane) is a yellow odorless pigment isolated from the rhizome of turmeric (*Curcuma longa* L.), reported to have strong anti-inflammatory, anticancer, and antioxidant properties. Anticarcinogenic and chemopreventive effects of curcurmin are attributed to its effect on several apoptotic molecules, transcription factors, cellular signaling pathways, and so forth. It has been found that topical application of curcurmin in TPA-pretreated skin of CD-1 mice significantly inhibited UVA induced ODC activity and ODC gene expression [152]. Further, the inhibitory effects of curcurmin were credited to its capacity to scavenge UVA induced ROS by several researchers [152, 153]. Oral and tropically doses of curcumin were equally potent to inhibit UV induced skin tumor in SKH-1 mice [154].

*In vitro* doses of curcumin have been reported to prevent UV induced apoptotic changes in human skin cancer (A431) cells, accompanied with release of cytochrome c and activation caspase-3. Moreover, UV induced ROS generation was also abolished by curcurmin [155]. In HaCaT cells curcurmin and UVR synergistically induced apoptotic cell death through activation of caspases 8, 3, and 9 and release of cytochrome c. Treatment with curcumin strongly inhibited COX-2 mRNA and protein expression in UVB irradiated HaCaT cell. Curcurmin inhibited UVB-induced AP-1 transcripcional activation and p38/JNK signaling pathway [156].

Phase I clinical trial of curcumin conducted by Chen et al. demonstrated that curcumin is not toxic to humans up to 8,000 mg/day when taken by mouth for 3 months and has potential effect in the cancer chemoprevention [157].

### 5.8. Ginger

Ginger (*Zingiber officinale Roscoe*) is one of the most heavily consumed dietary spices in the world. [6]-gingerol (oleoresin from the root of ginger), a major pharmacologically active component of ginger, has been shown to inhibit several cancers including skin cancer. This compound has antibacterial, anti-inflammatory, analgesic, and antitumor activity. It has been found to possess potent antioxidant activity. A study by Kim et al. [158] suggested that [6]-gingerol could be an effective therapeutic agent providing protection against UVB-induced skin disorders. Topical doses of gingerol prior to UVB irradiation in SKH-1 mice inhibited the induction of COX-2 mRNA and protein, as well as NF-*κ*B translocation.

In JB6 cells [6]-gingerol block EGF-induced cell transformation and inhibited EGF-induced AP-1 DNA binding activity [159].* In vitro*, pretreatment with gingerol reduced UVB-induced intracellular ROS levels, activation of caspases (3, 8, -9), and Fas expression. It also reduced UVR-induced expression and transactivation of COX-2 [158]. Translocation of NF-*κ*B from cytosol to nucleus in HaCaT cells was inhibited by [6]-gingerol via suppression of I*κ*B*α* phosphorylation.

### 5.9. Soy Isoflavone

Genistein, (4′,5,7-trihydroxyisoflavone) the most abundant isoflavone of the soy derived phytoestrogen compounds, is a potent antioxidant and inhibitor of tyrosine kinase activity. Genistein, daidzein (4′,7-dihydroxyisoflavone), and their 7-glycosides, genistin, and daidzin are the major isoflavonoids of soybeans and soy products. Soybean containing diet is associated with cancer chemoprevention. Genistein exerts its anti-initiational and promotional effects on skin carcinogenesis probably through blockage of DNA adduct formation and inhibition of oxidative (H_2_O_2_) and inflammatory (ODC activity) events in mouse skin [160]. In another study pretreatment of animals with 10 *μ*M of genistein 1 h prior to UVB exposure significantly inhibited UVB-induced H_2_O_2_ and MDA in skin and 8-OHdG in epidermis as well as internal organs [161]. Genistein protects the skin from long-term psoralen plus ultraviolet radiation induced skin damages [162]. Additionally, the inhibitory effect of soy isoflavones on TPA-induced cutaneous inflammation includes inhibition of proinflammatory cytokines, attenuation of oxidative stress, and activation of NF-*κ*B and expression of Cox-2 [163]. Moore et al. [164] studied genistein's photoprotective efficacy within the context of full thickness human reconstituted skin. Genistein (10, 20, and 50 *μ*M) 1 h prior to UVB (20 and 60 mJ/cm^2^) radiation inhibited UV-induced DNA damage and dose dependently preserved cutaneous proliferation and repair mechanics in skin samples.

### 5.10. Diallyl Sulphide (DAS)

DAS is an organosulfur compound derived from garlic (*Allium sativum* L.) and a few other genus of Allium plants and is well known to be beneficial for the prevention of lifestyle-related diseases. The therapeutic uses of garlic in cancer have been widely studied. Modulation in p53 and p21/waf1 expression by topical application of DAS was recorded in DMBA-induced mouse skin tumours [165, 166]. DMBA induced antiapoptotic proteins (survivin and bcl-2), oncoprotein (Ras), and signaling molecules activation (PI3K/Akt and MAPKs) were significantly downregulated by DAS supplementation [167]. Topical application of liposome-based formulation of DAS (250 microg/mouse), one hour after DMBA exposure, not only resulted in delay of tumorogenesis but also reduced the cumulative number and size of papillomas [168]. The pretreatment of DAS (10 mg/kg body-weight) showed 68.35% protection and posttreatment showed 59.49% protection, at an intermittent period of 48 h, against DMBA-induced DNA strand breakage in mouse skin [169].

## 6. Miscellaneous

Several other phytochemicals also have been reported for their imperative effects against skin cancer. These phytochemicals are enlisted in table with their respective sources and their mode of action in skin cancer prevention.

## 7. Conclusions

The phytochemicals possess antioxidant and antiinflammatory properties through which they contribute to their anticarcinogenic effects and to modulate various biochemical processes and molecular signaling pathways induced or mediated by carcinogens. Based on laboratory and epidemiological evidences it can be concluded that routine consumption/application of these natural agents may provide proficient safeguard against damaging effects of solar ultraviolet radiation as well as other skin carcinogens present in environment. Likewise, these natural agents in combination with sunscreens or skin care cosmetics may offer a rational approach for reducing skin cancers and other skin diseases.

## Figures and Tables

**Table 1:** 

Number	Name of phytochemicals	Rich source	Concerned pathways in skin cancer	References
1	Myricetin	Walnuts (*Juglans regia*)	Inhibits Akt activity to induce apoptosis (HaCaT cells) and also inhibits Fyn kinase activity	Kim et al. 2010 [58]; Jung et al. 2008 [59]
2	Caffeic acid	Coffee (*Coffea canephora*)	Inhibits Fyn kinase activity	Kang et al. 2009 [60]
3	Alpha-santalol	Sandalwood oil	Activates proapoptotic caspases and p53 and also cleave poly (ADP-ribose) polymerase through activating upstream caspase-8 and caspase-9.	Arasada et al. 2008 [61]
4	Nicotinamide	Peas, Asparagus, Mushroom, Squash	Repair UV induced DNA damage	Surjana et al. 2013 [62]
5	Norathyriol	Mango, *Hypericum elegans,Tripterospermum lanceolatum*, and so forth	Inhibit AKT activation, epidermal growth factor (EGF), and ERk1/2 to attenuate UVB-induced phosphorylation in MAPK signaling	Li et al. 2012 [63]
6	Retinoids (Acitretin and isotretinoin)	Vitamin A rich food source like Carrot, Spinach, pumpkin, and so forth	Prevention of nonmelanoma skin cancer isotretinoin is preferred in xeroderma pigmentosum and nevoid basal cell carcinoma syndrome, whereas acitretin is more used in transplant recipients, psoriasis, and severe sun damage	Bettoli et al. 2013 [64]
7	Shikonin	*Lithospermum erythrorhizon *	Reverse AMPK activity in TPA induced suppression	Li et al., 2012 [65]
8	Ferulic acid (FA)	*Rich source in Phaseolus vulgaris*, Rice and Maize Bran	Inhibit MMP-2 and MMP-9 protein expression	Staniforth et al. 2012 [66]
9	Honokiol	Magnolia plant	Inhibit UVB-induced expression of cyclooxygenase-2, prostaglandin E(2), PCNA, and proinflammatory cytokines (TNF-*α*, IL-1*β* and IL-6)	Vaid et al. 2010 [67]
10	Pycnogenol B7	French maritime pine, *Pinus pinaster *	Offered protection against sun UV induced acute inflammation, immunosuppression, and carcinogenesis	Sime and Reeve, 2004 [68]
11	Guggulsterone	*Commiphora mukul *	attenuates Cox-2 and i NOS protein expression	Sarfaraz et al., 2008 [69]
12	Humulone	*Humulus lupulus *	Inhibit TPA-induced epidermal COX-2 expression by blocking upstream kinases IKK and JNK and activates NF-kappaB and AP-1.	Lee et al., 2007 [70]
13	Apigenin	Parsley and Onions	Inhibits UVB-induced COX-2 expression by targeting src Kinase	Byun et al., 2012 [71]
14	5-Hydroxy-3,6,7,8,3′,4′-hexamethoxyflavone (5-OH-HxMF),	Fruits of citrus genus specially Peels of sweet orange	Inhibitory effects on TPA-induced expression of iNOS and COX-2 in mouse skin and activates NF-*κ*B and STAT3	Lai et al., 2007 [72]
15	3,3′-Diindolylmethane (DIM)	Cruciferous vegetables like broccoli, cabbage, and so forth	Inhibited the TPA-induced increases in COX-2, iNOS, chemokine (C-X-C motif) ligand (CXCL) 5, IL-6, NF-*κ*B, IKK, and ERK-1/2	Kim et al., 2010 [73]
16	Xanthorrhizol	*Curcuma xanthorrhiza *	Reduces ODC, iNOS, and COX-2 level, regulated by the NF-*κ*B, MAPK/Akt	Chung et al., 2007 [74]
17	Oleandrin	*Nerium oleander *	Reverts PI3K, Akt phosphorylation, and activation of NF-*κ*B	Afaq et al., 2004 [75]
18	Delphinidin	genus* Delphinium *	Decreases cell viability and PCNA expression, peroxidation, and formation of 8-OHdG in HaCaT cells and SKH-1 animals	Afaq et al., 2007 [76]
